# Does Treating Adult ADHD Reduce GP Mental Health Workload?

**DOI:** 10.1192/bjo.2026.11544

**Published:** 2026-06-30

**Authors:** Richard Bunn, Jonathan Hunter, James Gilliland, Adrian McCauley, Sarah O'Hagan

**Affiliations:** 1 NHS, Belfast, United Kingdom; 2 GP Practice, Belfast, United Kingdom

## Abstract

**Aims:**

To determine if treating ADHD in a busy inner city GP practice reduces the workload of GP’S and reduces frequency of missed appointments

**Methods:**

Data was collected from a GP surgery in Belfast which prescribes ADHD medication according to Shared Care Guidelines. Patients were excluded if they had been on ADHD medication for less than 12 months (as of November 2024) or if they had received a diagnosis from childhood. 19 Patients were found to fit the criteria. Data was collected to compare the number of missed appointments pre vs post ADHD diagnosis and treatment, as well as comparing the number of consultations which patients required related to their mental health.

**Results:**

Results from this Audit found that there was no significant difference in the number of appointments which patients missed in the 12 months before ADHD treatment and diagnosis compared to after. On the other hand, it was found that the number of GP Consultations related to Mental Health almost halved (37 to 19) in the 12 months after ADHD treatment and diagnosis compared to the 12 months before.

**Conclusion:**

Findings indicate that the diagnosis and treatment of patients with ADHD, reduces the burden which they place on Primary Care services, not only in time spent with the GP but also contacting receptionist, practice nurse prescriptions etc, never mind the benefits for the patient and their loved ones.

